# Quantitative Prediction of Coal–Gangue Content Using Terahertz Time-Domain Spectroscopy and Physics-Informed Machine Learning

**DOI:** 10.3390/ma19173776

**Published:** 2026-09-04

**Authors:** Zeping Liu, Lipeng Hu, Jianfei Xu, Yadong Yang, Sitong Li, Zhou Xu, Longhai Liu, Jiabao Li, Houli Liu, Dongdong Ye

**Affiliations:** 1 State Key Laboratory of Intelligent Mining Equipment Technology, Taiyuan 030032, China; 2 Shanxi TZCO Intelligent Mining Equipment Technology Co., Ltd., Taiyuan 030032, China; 3 School of Artificial Intelligence, Anhui Polytechnic University, Wuhu 241000, China; 4 Key Laboratory of Pressure System and Safety, Ministry of Education, School of Mechanical and Power Engineering, East China University of Science and Technology, No. 130 Meilong Road, Shanghai 200237, China; 5 School of Intelligent Manufacturing, Wuhu University, Wuhu 241008, China; 6 School of Electrical and Automation, Wuhu Vocational Technical University, Wuhu 241006, China; 7 Key Laboratory of Opto-Electronics Information Technology, Ministry of Education, School of Precision Instruments and Opto-Electronics Engineering, Tianjin University, Tianjin 300072, China; 8 Aviation Industry Corporation Huadong Photoelectric Co., Ltd., Wuhu 241003, China; 9 State Key Laboratory of Digital Intelligent Technology for Unmanned Coal Mining, Anhui University of Science and Technology, Huainan 232001, China

**Keywords:** coal–gangue separation, terahertz time-domain spectroscopy, machine learning, physics-informed neural network, effective medium theory

## Abstract

Quantitative determination of gangue content is important for efficient coal use and intelligent coal–gangue separation. We combine transmission terahertz time-domain spectroscopy (THz-TDS), multidomain feature fusion, and machine learning to predict gangue mass fraction in coal–gangue mixtures. Time- and frequency-domain signals, refractive index, absorption and extinction coefficients, and complex permittivity were extracted from samples with different gangue contents. Five-fold cross-validation was used to compare random forest, support vector regression, Gaussian process regression, an artificial neural network, and an Effective Medium Theory-constrained Physics-Informed Neural Network (EMT-PINN). EMT-PINN achieved the best performance, with a coefficient of determination (R^2^) of 0.81 ± 0.15, a mean absolute error (MAE) 3.17 ± 0.59%, and a root mean square error (RMSE) of 5.79 ± 0.21%, compared with R^2^ values of 0.72 ± 0.08, 0.61 ± 0.21, 0.74 ± 0.11, and 0.64 ± 0.18 for RF, SVR, GPR, and ANN, respectively. These results demonstrate the potential of physics-informed THz spectroscopy for rapid and physically interpretable quantitative characterization of coal–gangue mixtures.

## 1. Introduction

Against the background of global climate action and China’s carbon-neutrality and sustainable-development strategies, the transition toward a low-carbon energy system requires more efficient and cleaner use of existing energy resources. Although the long-term transition emphasizes low-carbon energy sources, coal remains an important energy resource, making efficient coal utilization and resource recovery relevant to reducing resource waste and associated emissions [1,2]. Coal–gangue is a solid waste generated during coal mining, preparation, and processing. Large accumulations occupy land, lower the calorific value and utilization efficiency of coal, increase transportation costs and equipment wear, and may undergo spontaneous combustion that releases harmful gases [3,4,5]. Efficient identification of coal and gangue is therefore essential to coal quality and resource utilization. Conventional separation depends heavily on visual inspection and operator experience, resulting in low efficiency and safety risks that are incompatible with green and intelligent mining [6].

Terahertz time-domain spectroscopy (THz-TDS) is an emerging detection technique that overcomes several limitations of conventional spectroscopy [7,8] and has shown particular promise for mineral identification. Terahertz radiation lies between the microwave and infrared regions and has low photon energy, strong penetration through many nonconductive materials, and sensitivity to dielectric and structural properties. THz-TDS directly measures the dielectric response of a specimen and has been widely used in materials science [9], biomedicine [10], and security inspection [11]. Its feasibility for distinguishing coal from gangue has also been explored [12,13,14].

Combining THz-TDS with machine learning has become an effective route to improving coal–gangue recognition. Ye et al. [15] combined THz-TDS with support vector machines, artificial neural networks, and random forests and obtained classification accuracies above 96% on a test set. Liu et al. [16] developed a three-channel convolutional neural network using absorption coefficient, refractive index, and their slopes, achieving 97.62% accuracy for lignite, bituminous coal, and anthracite. Miao et al. [17] combined THz-TDS with principal component and cluster analyses and reported complete discrimination among several bituminous coals using absorption spectra.

These studies largely rely on purely data-driven models whose performance depends strongly on the quantity and quality of labeled data. Large labeled datasets are expensive and time-consuming to obtain under mining conditions, whereas small datasets increase the risk of overfitting and poor generalization. Physics-Informed Neural Network (PINN) provides a way to introduce physical prior knowledge into data-driven learning by embedding governing relations in the loss function [18]. In spectral reconstruction, Wu et al. [19] used a PINN to enforce the physical consistency of the image-formation process and improve generalization under changing illumination. For THz analysis of coal–gangue mixtures, changes in composition produce systematic changes in effective dielectric properties. Effective Medium Theory (EMT) can describe the relation between component fraction and mixture permittivity. Embedding EMT in a PINN may therefore combine spectral features with a physically meaningful dielectric constraint for quantitative gangue-content prediction.

Although THz-TDS combined with machine learning has shown promising performance for coal–gangue recognition, existing studies mainly focus on qualitative classification using purely data-driven models. Quantitative prediction of gangue content based on THz spectroscopy remains insufficiently explored, particularly when only limited experimental data are available. Furthermore, the potential of incorporating physical prior knowledge into THz-based regression models has received little attention. To address these limitations, this study proposes an Effective Medium Theory (EMT)-constrained Physics-Informed Neural Network (EMT-PINN), in which the dielectric mixing relationship is embedded into the learning process as a physical constraint. By integrating multidomain THz features with physically meaningful dielectric information, the proposed framework aims to improve both prediction accuracy and physical interpretability for quantitative gangue-content prediction.

## 2. Experiments and Methods

### 2.1. Experimental Setup

**Figure 1:**
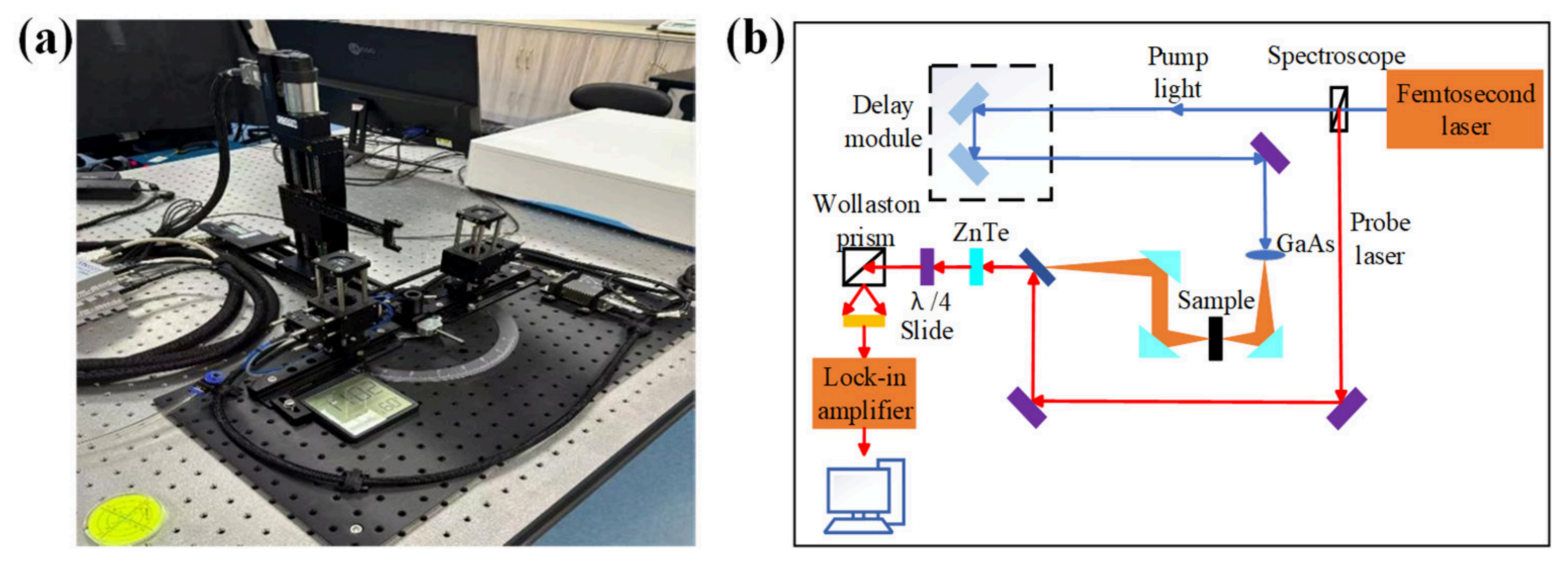
(**a**) TeraSmart terahertz time-domain spectrometer; (**b**) operating principle of transmission THz-TDS.

Figure 1a shows the TeraSmart THz-TDS system (Menlo Systems GmbH, Martinsried, Germany). According to the product and user manuals, the system provides a spectral range above 6 THz, a dynamic range above 100 dB, a time-window exceeding 850 ps, and a frequency resolution below 1.2 GHz. The laser operates at a repetition rate of 100 MHz with a 1560 nm fiber-coupled output and a pulse width below 90 fs. The recommended optical power and bias voltage for the Tera15-HP-TX emitter are 40–45 mW and 200 V, respectively; the receiver requires no bias. Measurements were performed at 15–35 °C with controlled humidity, and the THz beam path was purged with dry nitrogen to suppress water-vapor absorption.

The transmission geometry is illustrated in Figure 1b. Broadband THz pulses generated by a femtosecond-laser-driven photoconductive source propagate through the optical path and interact with the specimen. Absorption, transmission, and reflection depend on the physical, chemical, and structural properties of the material. After transmission through the specimen, the detector records the changes in pulse amplitude and phase, from which the time-domain waveform, spectrum, and optical parameters are obtained.

### 2.2. Sample Preparation

Thin pellets made only from coal and gangue powders were mechanically fragile. High-density polyethylene (PE) powder was therefore used as a binder and dilution matrix. PE has low absorption and a stable refractive index in the THz band, which reduces scattering, preserves the optical response of coal and gangue, and improves pellet strength [20].

The coal and gangue powders were certified reference materials obtained through China’s National Sharing Platform for Reference Materials. The coal was GBW11103, an anthracite reference material prepared by the Testing Center of the China Coal Research Institute and jointly characterized by national and provincial testing laboratories. The gangue was GSB06-2182-2008 (ZBM110A), supplied by Jinan Zhongbiao Technology Co., Ltd. (Jinan, China). Both materials were stored in a dry, ventilated environment and thoroughly mixed before use. Certified values and uncertainties were taken from the corresponding certificates.

**Figure 2:**
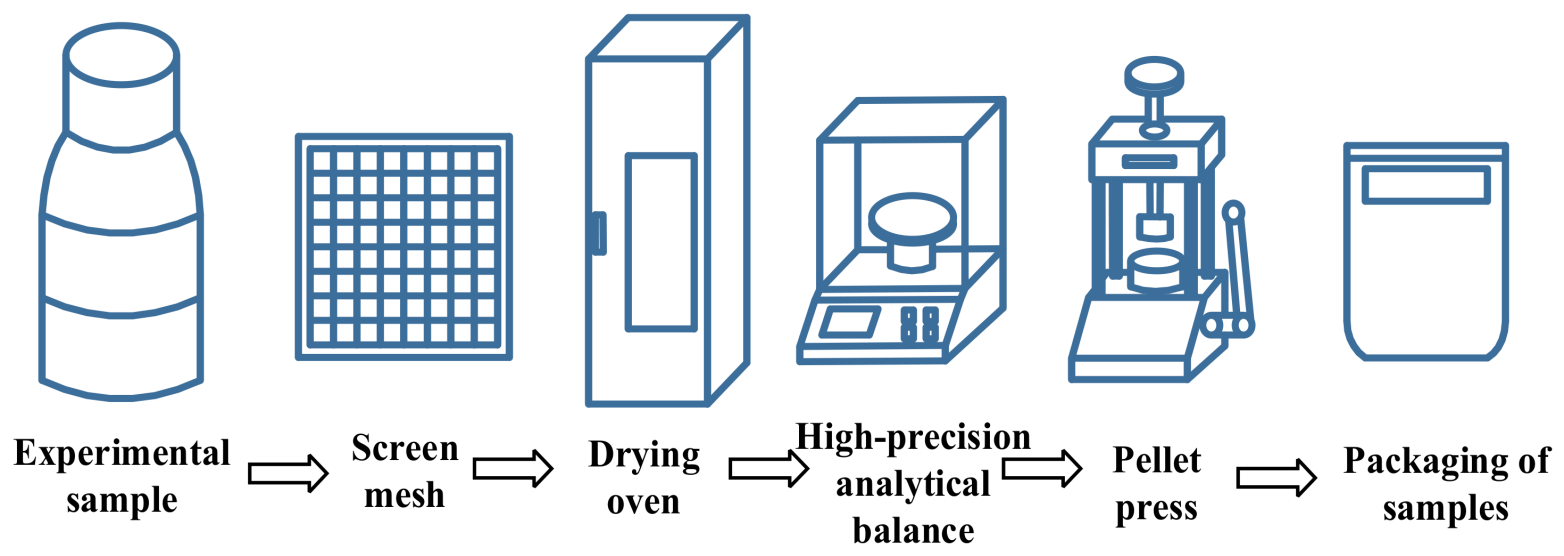
Preparation procedure for the pressed-pellet specimens.

The pellet-preparation procedure is shown in Figure 2. Coal, gangue, and PE powders were passed separately through a 74 μm sieve to obtain a consistent particle-size range. The sieved powders were then dried at 80 °C for 4–5 h to remove adsorbed moisture and reduce humidity-related variation in THz propagation. After drying, the coal powder and gangue powder were weighed using a high-precision analytical balance and mixed according to a predetermined ratio. Considering the variation range of gangue content in actual coal mining processes, this study designed eight samples with different gangue contents—0%, 5%, 10%, 15%, 20%, 25%, 30%, and 100%—to analyze the impact of varying coal-to-gangue ratios on terahertz (THz) spectral response characteristics. The gangue content is defined as the percentage of gangue mass relative to the total mass of the coal–gangue mixture. To enhance the model’s ability to detect subtle changes in low gangue content, which is common in practical coal production (typically 5–30%), the samples within this range were further subdivided into finer gradients. The specific mixing ratios are shown in Table 1.

To improve mechanical stability and measurement uniformity, each coal–gangue mixture was blended with PE at a 1:1 mass ratio and ground until the components were uniformly dispersed. The powder was loaded into a 10 mm diameter die and pressed at 10 MPa for 5 min, producing pellets approximately 1.1 mm thick. The actual thickness of each pellet was measured, and the specimen was labeled and sealed with its gangue-content information.

Five replicate pellets were prepared for each gangue content, yielding 40 specimens. Each pellet was measured five times by THz-TDS, providing 200 time-domain datasets. Repeated measurements were averaged to reduce instrument noise, local specimen heterogeneity, and operator-induced variation.

During measurement, the temperature was held stable and dry gas was supplied to the optical path to maintain the relative humidity below 1%. These procedures improved comparability among specimens and provided a consistent basis for feature extraction, feature fusion, and regression modeling.

### 2.3. Extraction of Optical Parameters

**Figure 3:**
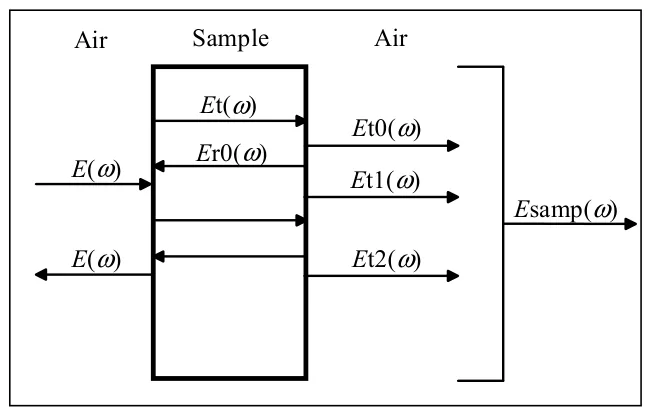
Multiple reflection and transmission of a THz wave through a plane-parallel specimen.

Optical parameters were derived from the transmission THz-TDS measurements. Figure 3 illustrates propagation through a plane-parallel specimen. When a THz field *E*(*ω*) enters the specimen from air, multiple reflections and transmissions occur according to the Fresnel relations [21,22]. The reflected and transmitted fields are denoted by *E*_r_(*ω*) and *E*_t_(*ω*), and the fields after m internal reflections are denoted by *E*_rm_(*ω*) and *E*_tm_(*ω*), respectively.

Following the methods of Duvillaret et al. [23] and Dorney et al. [24], the reference field propagating in air is *E*_ref_(*ω*), the field transmitted through the specimen is *E*_samp_(*ω*), and the complex transmission coefficient is

(1) $$T(\omega )=\frac{E_{\mathrm{samp}}(\omega )}{E_{\mathrm{ref}}(\omega )}$$

The refractive index n(*ω*), extinction coefficient k(*ω*), and absorption coefficient α(*ω*) are calculated as

(2) $$n(\omega )=1+\frac{\varphi (\omega )c}{\omega d}$$

(3) $$k(\omega )=\frac{c}{\omega d}\ln\frac{\left|E_{\mathrm{ref}\;}(\omega )\right|}{\left|E_{\mathrm{samp}\;}(\omega )\right|}$$

(4) $$\alpha (\omega )=\frac{2}{d}\ln\frac{\left|E_{\mathrm{ref}\;}(\omega )\right|}{\left|E_{\mathrm{samp}\;}(\omega )\right|}$$

where *φ*(*ω*) is the phase difference between the specimen and reference signals, c is the speed of light in vacuum, and d is the specimen thickness. The complex relative permittivity is then calculated as

(5) $$\hat{\varepsilon }=\varepsilon_{r}+i\varepsilon_{i}=n^{2}(\omega )-k^{2}(\omega )+i2n(\omega )k(\omega )$$

where *ε_r_* and *ε_i_* are the real and imaginary parts of the complex permittivity, respectively.

### 2.4. Machine-Learning Models

Although pure gangue (100%) specimens were also prepared as reference samples to characterize the intrinsic dielectric properties of gangue, only specimens with 0–30% gangue content were used for quantitative gangue-content prediction. Within this range, coal was considered the host medium and gangue the dispersed phase, making the Maxwell–Garnett effective-medium model suitable for imposing the dielectric mixing constraint. PE was added at a fixed 1:1 mass ratio to all coal–gangue mixtures and was therefore not treated as an independent variable in the gangue-content prediction. Accordingly, the EMT constraint was used to describe the composition-dependent dielectric evolution of the coal–gangue system, while the fixed PE contribution was regarded as a common matrix effect across all specimens.

Because pure coal and pure gangue specimens were experimentally characterized, conventional EMT-based inversion is feasible, in principle. However, such inversion relies mainly on the effective permittivity, whereas the proposed EMT-PINN incorporates the EMT relation as a physical constraint while simultaneously using multidomain THz features. However, such inversion primarily relies on the effective complex permittivity to infer the mixture composition. In the proposed EMT-PINN, the measured THz response is represented by multidomain features, including time-domain characteristics of pulse delay and attenuation, frequency-domain characteristics of spectral amplitude and energy distribution, and optical parameters describing the electromagnetic response. These features provide complementary representations of the same THz measurement, while the measured complex permittivity is additionally used to construct the EMT-based physics loss. Thus, EMT-PINN does not introduce an independent information source beyond THz-TDS; rather, it jointly exploits multidomain THz observables for data fitting and the dielectric mixing relationship for physical regularization. This design combines the richer feature representation of data-driven regression with the physical consistency imposed by EMT.

Time-domain, frequency-domain, and optical-parameter features were extracted and evaluated by five-fold cross-validation. Five-fold cross-validation was selected to provide a balance between training-data utilization and reliable validation for the relatively limited dataset, while avoiding excessive reduction of the training set. The dataset was divided into five folds, with four folds (80%) used for training and the remaining fold (20%) used for validation in each iteration. Each sample was used once for validation, and the reported performance metrics were calculated from the five validation folds. Random Forest (RF), Support Vector Regression (SVR), Gaussian Process Regression (GPR), Artificial Neural Network (ANN) and EMT-constrained PINN (EMT-PINN) regression models were compared using the coefficient of determination (R^2^), Root Mean Square Error (RMSE), and Mean Absolute Error (MAE).

To ensure reproducibility, the hyperparameters of all conventional machine learning models were configured based on commonly used values in the literature and preliminary pilot experiments, combined with the same five-fold cross-validation strategy for performance evaluation. Table 2 summarizes the key hyperparameters for each model. Specifically, RF used 100 trees and a minimum leaf size of 4; SVR employed a radial basis function (RBF) kernel with a regularization parameter C = 10 and gamma = 0.1; GPR utilized an automatic relevance determination squared exponential (ARD-SE) kernel; and the ANN consisted of two hidden layers (20 and 10 neurons) with ReLU activation, trained using the Adam optimizer with a learning rate of 0.001 and a batch size of 16.

#### 2.4.1. Random Forest (RF)

**Figure 4:**
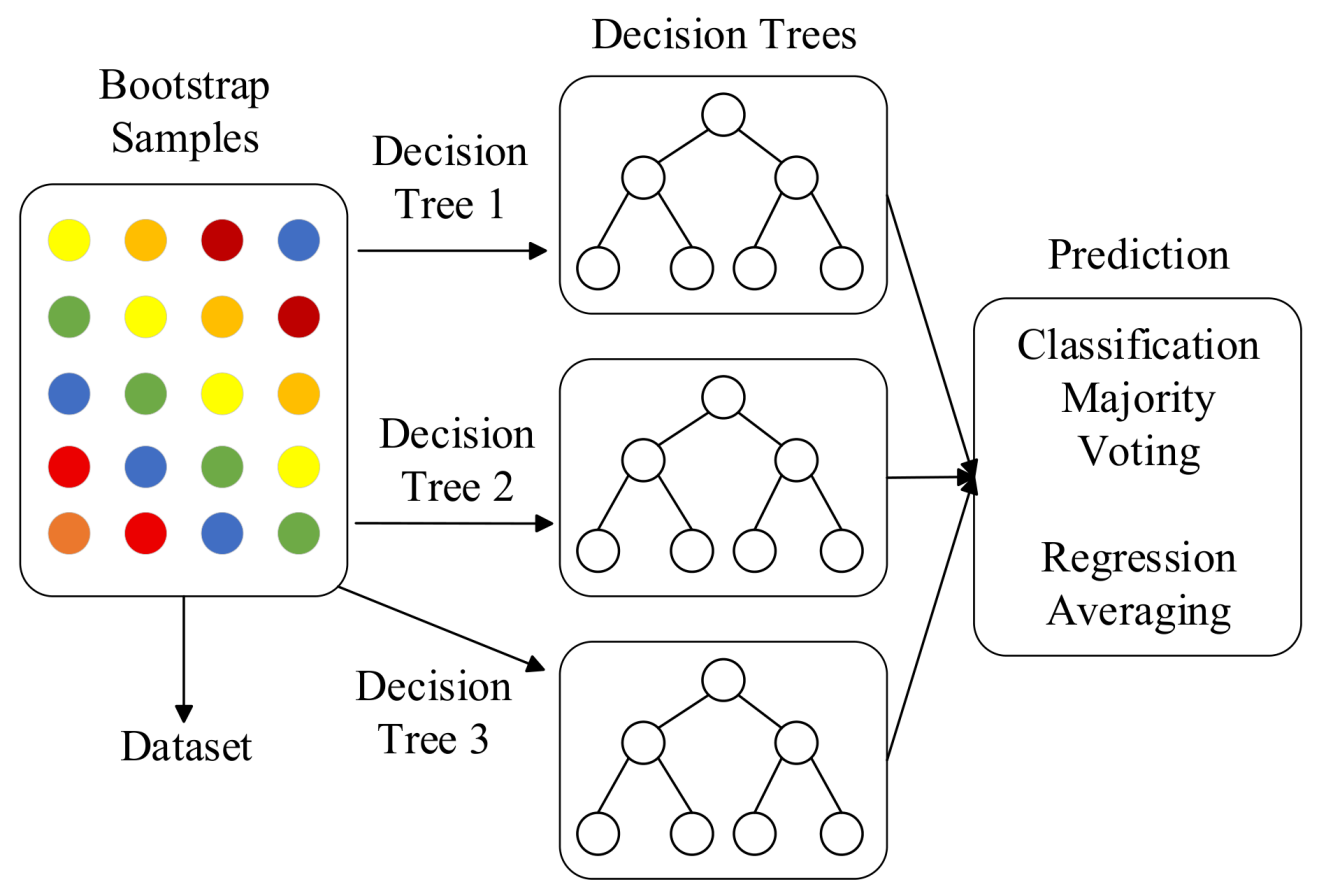
Workflow of the random-forest regression model.

RF is an ensemble learning method that combines multiple decision trees for performing regression by aggregating their individual predictions [25]. Multiple training subsets are generated by bootstrap sampling, and a decision-tree regressor is fitted to each subset. The number of trees was selected to provide stable ensemble predictions while maintaining reasonable computational complexity. The final prediction is the average output of the trees. Ensemble averaging reduces overfitting and provides robust nonlinear mapping for complex spectral data. The RF workflow is shown in Figure 4.

#### 2.4.2. Support Vector Regression (SVR)

**Figure 5:**
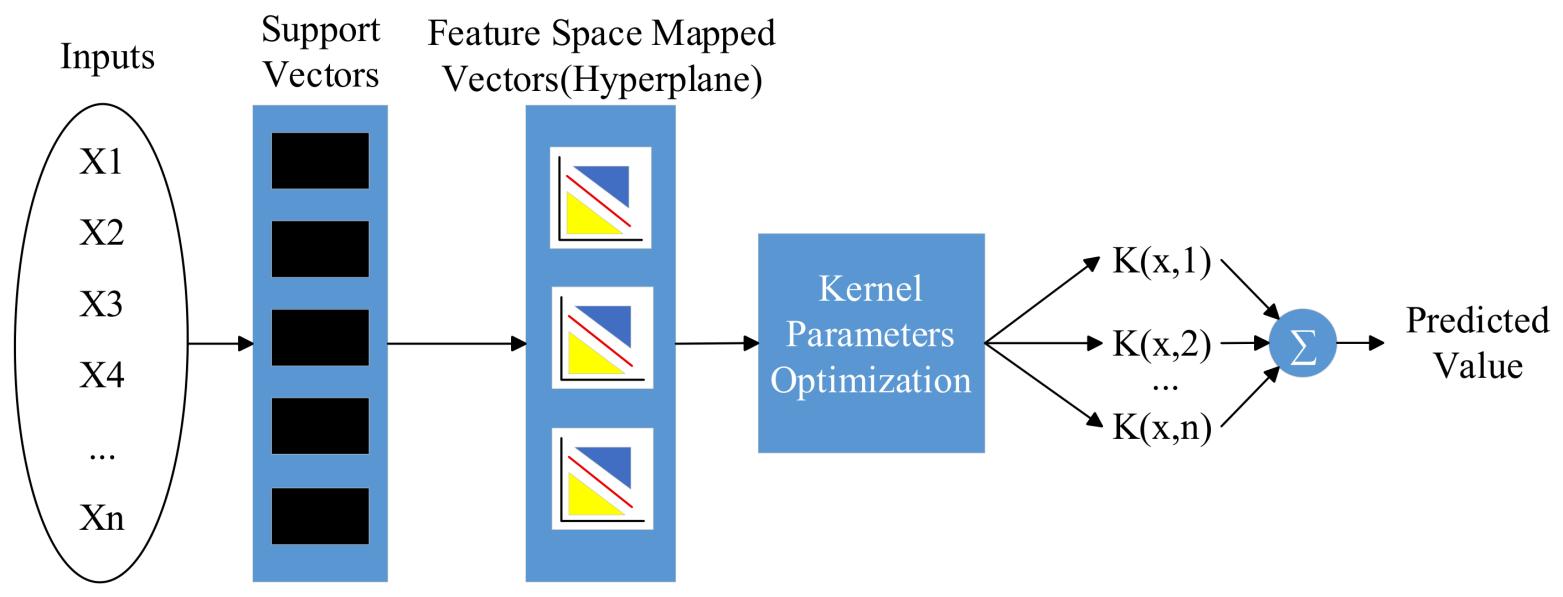
Workflow of the support-vector-regression model.

Support Vector Regression (SVR) extends the support vector machine framework to regression problems by constructing a function that minimizes prediction error while controlling model complexity [26]. SVR applies structural-risk minimization to balance fitting accuracy and generalization. A kernel maps the input features into a high-dimensional space, where an optimal regression hyperplane is obtained using an ε-insensitive loss. A Gaussian radial-basis-function kernel (RBF) was selected because it can model nonlinear relationships between the THz-derived features and gangue content. The SVR workflow is shown in Figure 5.

#### 2.4.3. Gaussian Process Regression (GPR)

**Figure 6:**
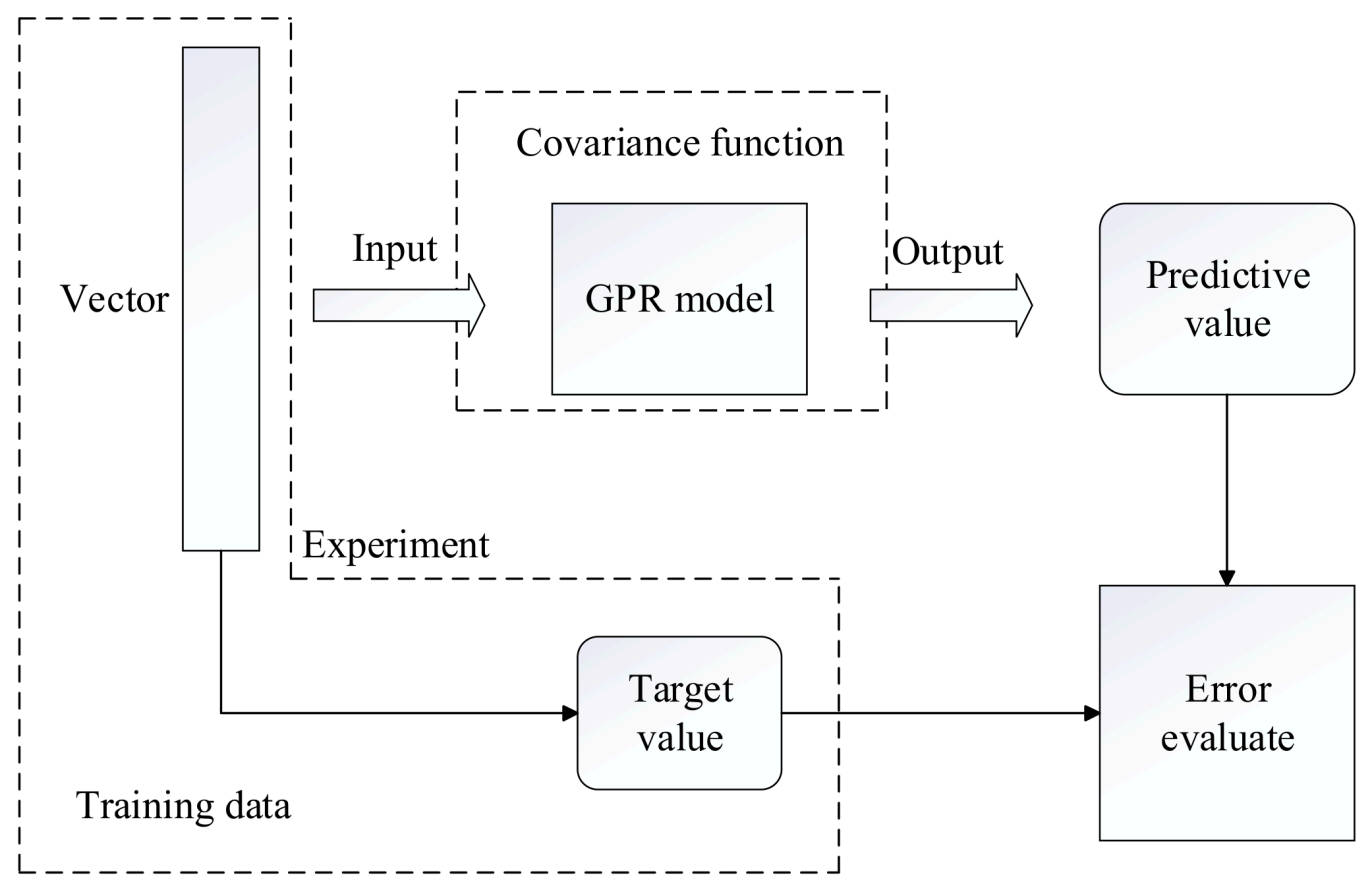
Workflow of the Gaussian-process-regression model.

GPR is a Bayesian nonparametric regression method that treats the unknown regression function as a Gaussian process and describes sample similarity through a covariance function [27]. GPR was adopted because its probabilistic formulation provides both nonlinear regression capability and an estimate of prediction uncertainty. An automatic-relevance-determination squared-exponential kernel was used to learn the relative importance of the input features. The GPR workflow is shown in Figure 6.

#### 2.4.4. Artificial Neural Network (ANN)

**Figure 7:**
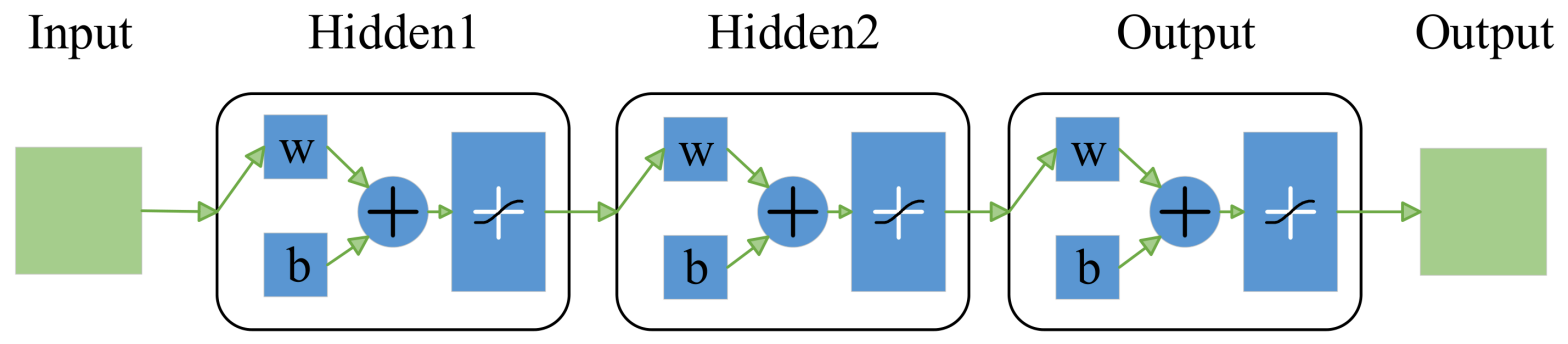
Architecture and training workflow of the artificial neural network.

The ANN is a multilayer feed-forward network trained by error backpropagation [28]. It contains two hidden layers with 20 and 10 neurons. The 20-neuron hidden layer was selected to provide sufficient nonlinear representation capacity while limiting model complexity and the risk of overfitting, given the limited number of training samples. Weights and biases were iteratively adjusted to minimize the error between predicted and measured gangue contents. This architecture provides nonlinear fitting capacity for the coupled relation between THz features and mixture composition. The ANN workflow is shown in Figure 7.

#### 2.4.5. Physics-Informed Neural Network (PINN)

A PINN combines data fitting with constraints derived from physical laws. The governing relation is included as a regularization term in the loss function, producing a joint objective that contains data and physics losses. This strategy can reduce the dependence of conventional machine learning on large datasets, prevent predictions from drifting away from physically admissible behavior, and improve generalization under small-sample conditions [29].

**Figure 8:**
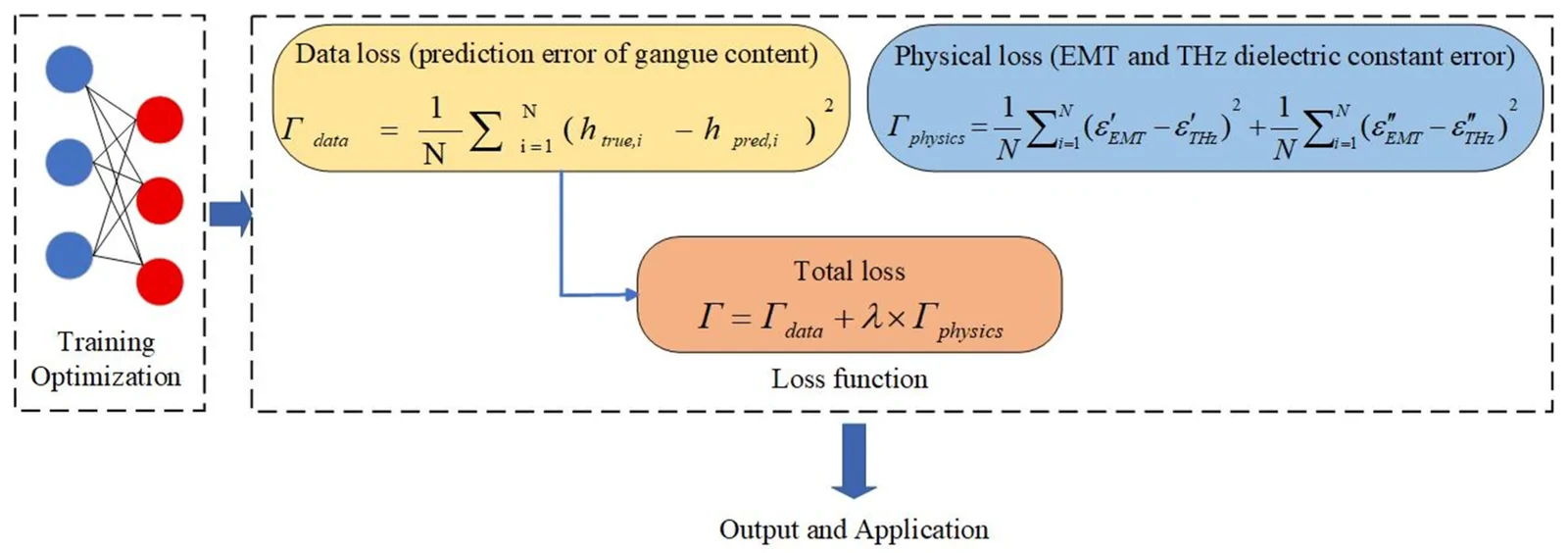
Architecture and loss construction of the EMT-constrained physics-informed neural network.

Conventional regression learns only a statistical mapping between input features and target values. Unlike conventional PINNs that employ governing differential equations as physical constraints, the proposed EMT-PINN incorporates the dielectric mixing relationship described by Effective Medium Theory into the network loss function. Consequently, the predicted gangue content is constrained not only by experimental observations, but also by the intrinsic electromagnetic behavior of coal–gangue mixtures. To introduce prior knowledge of the coal–gangue mixture, we embedded an EMT constraint in a deep neural network, forming the EMT-PINN. The EMT constraint was introduced to incorporate prior dielectric-mixture knowledge into the data-driven learning process, thereby improving physical consistency and reducing reliance on purely empirical correlations. The model jointly learns from the measured data and the dielectric mixing relation. Figure 8 summarizes the constraint and training workflow.

The data loss was defined by the mean square error

(6) $$\Gamma_{data}=\frac{1}{N}{\sum_{i=1}^{N}\left(h_{true,i}-h_{pred,i}\right)}^{2}$$

where *h_true_*_,_*_i_* and *h_pred_*_,_*_i_* are the measured and predicted gangue contents, respectively. The predicted gangue fraction f was then substituted into the Maxwell–Garnett effective-medium model to calculate the theoretical complex permittivity of the mixture:

(7) $$\varepsilon_{eff}=\varepsilon_{c}\frac{\varepsilon_{g}+2\varepsilon_{c}+2f(\varepsilon_{g}-\varepsilon_{c})}{\varepsilon_{g}+2\varepsilon_{c}-f(\varepsilon_{g}-\varepsilon_{c})}$$

where *f* is the gangue mass fraction, $\varepsilon_{c}$ and $\varepsilon_{g}$ are the complex permittivities of coal and gangue, respectively, and $\varepsilon_{\mathrm{eff}}$ is the effective complex permittivity of the mixture. The difference between the EMT prediction and the measured complex permittivity defines the physics loss:

(8) $$\Gamma_{physics}=\frac{1}{N}{\sum_{i=1}^{N}\left({\varepsilon^{\prime }}_{EMT}-{\varepsilon^{\prime }}_{THz}\right)}^{2}+\frac{1}{N}{\sum_{i=1}^{N}\left({\varepsilon^{\prime \prime }}_{EMT}-{\varepsilon^{\prime \prime }}_{THz}\right)}^{2}$$

where $\frac{1}{N}\sum_{i=1}^{N}\left(\varepsilon_{\mathrm{EMT}}^{\prime }\;-{\;\varepsilon }_{\mathrm{THz}}^{\prime }\right)$ ^2^ and $\frac{1}{N}\sum_{i=1}^{N}\left(\varepsilon_{\mathrm{EMT}}^{\prime \prime }\;-{\;\varepsilon }_{\mathrm{THz}}^{\prime \prime }\right)$ ^2^ are the mean square errors for the real and imaginary parts of the permittivity. The total EMT-PINN loss is

(9) $$\Gamma =\Gamma_{data}+\lambda \times \Gamma_{physics}$$

where λ is the physics-loss weight that balances data fitting and physical consistency. The network parameters were optimized using the Adaptive Moment Estimation (Adam) optimization algorithm, so that the predictions simultaneously fitted the measured gangue contents and satisfied the EMT relation. Compared with a conventional backpropagation network, this constraint is intended to improve generalization and physical interpretability when only a small dataset is available.

## 3. Results and Discussion

### 3.1. THz-TDS Signal Characteristics

**Figure 9:**
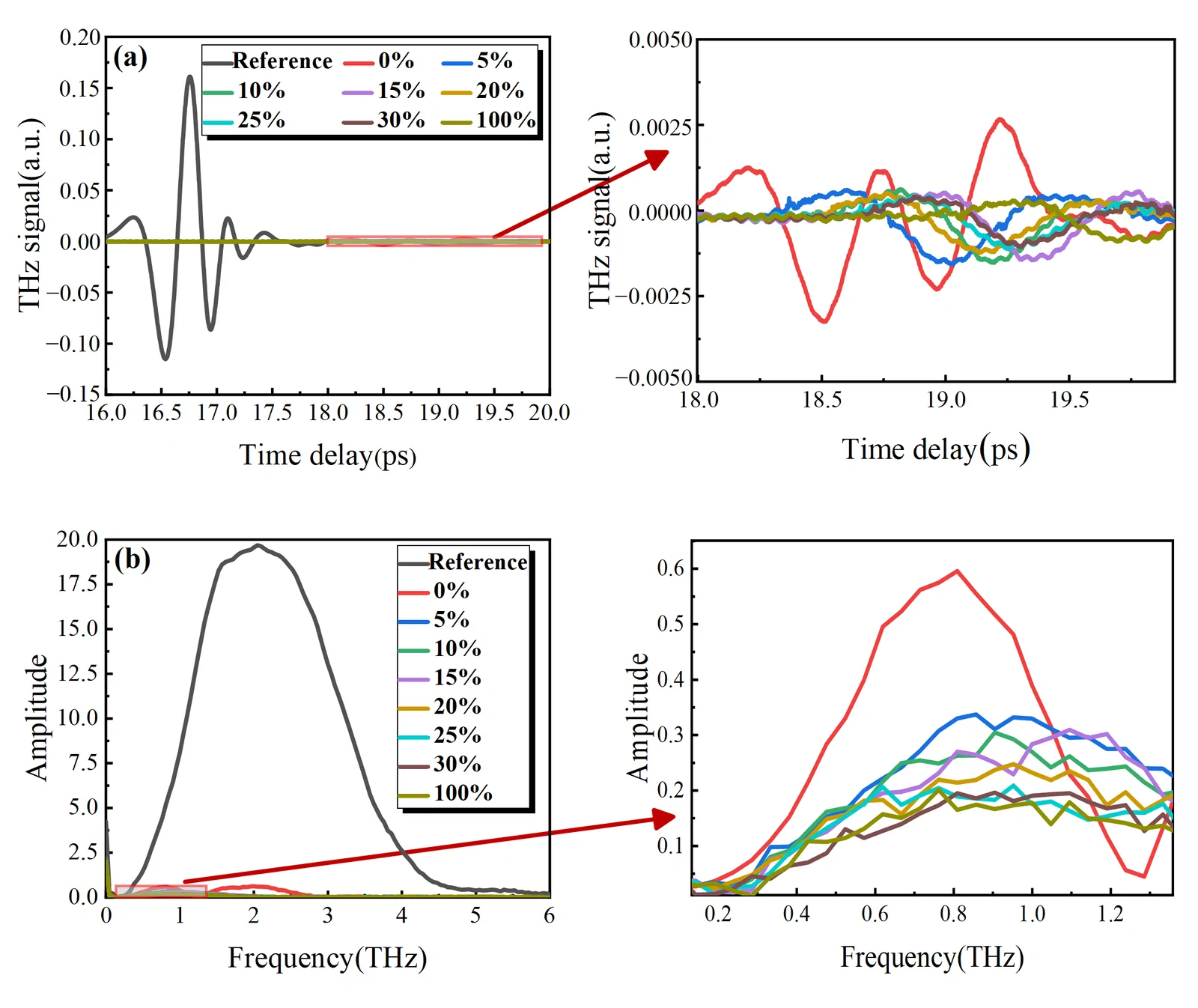
THz responses of specimens with different gangue contents: (**a**) time-domain waveforms; (**b**) frequency-domain spectra.

Transmission THz-TDS was used to measure the time-domain response of each coal–gangue mixture. Figure 9a shows that all specimens retained a pulse shape similar to that of the reference, while increasing gangue content caused clear amplitude attenuation and temporal delay. The reference peak occurred at approximately 16.75 ps, whereas the specimen peaks were distributed between 18.3 and 19.5 ps, and shifted progressively to later times as the gangue fraction increased. Because coal and gangue have different dielectric responses, the effective refractive index of the mixture increases with gangue content, reducing the THz propagation velocity. The resulting delay is therefore sensitive to mixture composition.

The transmitted amplitude also decreased with increasing gangue content, with the largest contrast observed between pure coal and high-gangue specimens. Mineral components in gangue, including quartz and clay minerals, produce dielectric loss and scattering in the THz band. Increasing their fraction raises the electromagnetic energy dissipation in the heterogeneous mixture. Time-domain attenuation thus provides another indicator of gangue content.

Fast Fourier transformation of the time-domain signals produced the spectra in Figure 9b. The reference covered approximately 0–6 THz, while the useful specimen response was concentrated below 3 THz and exhibited a prominent feature near 0.6–0.8 THz. Relative to the reference, all specimen spectra were attenuated, and the attenuation increased with gangue content. Changes in composition modify the dielectric response and loss mechanisms associated with polarization, lattice motion, and charge-carrier dynamics, thereby redistributing spectral energy. Frequency-domain features consequently complement the propagation delay and attenuation captured in the time domain.

**Figure 10:**
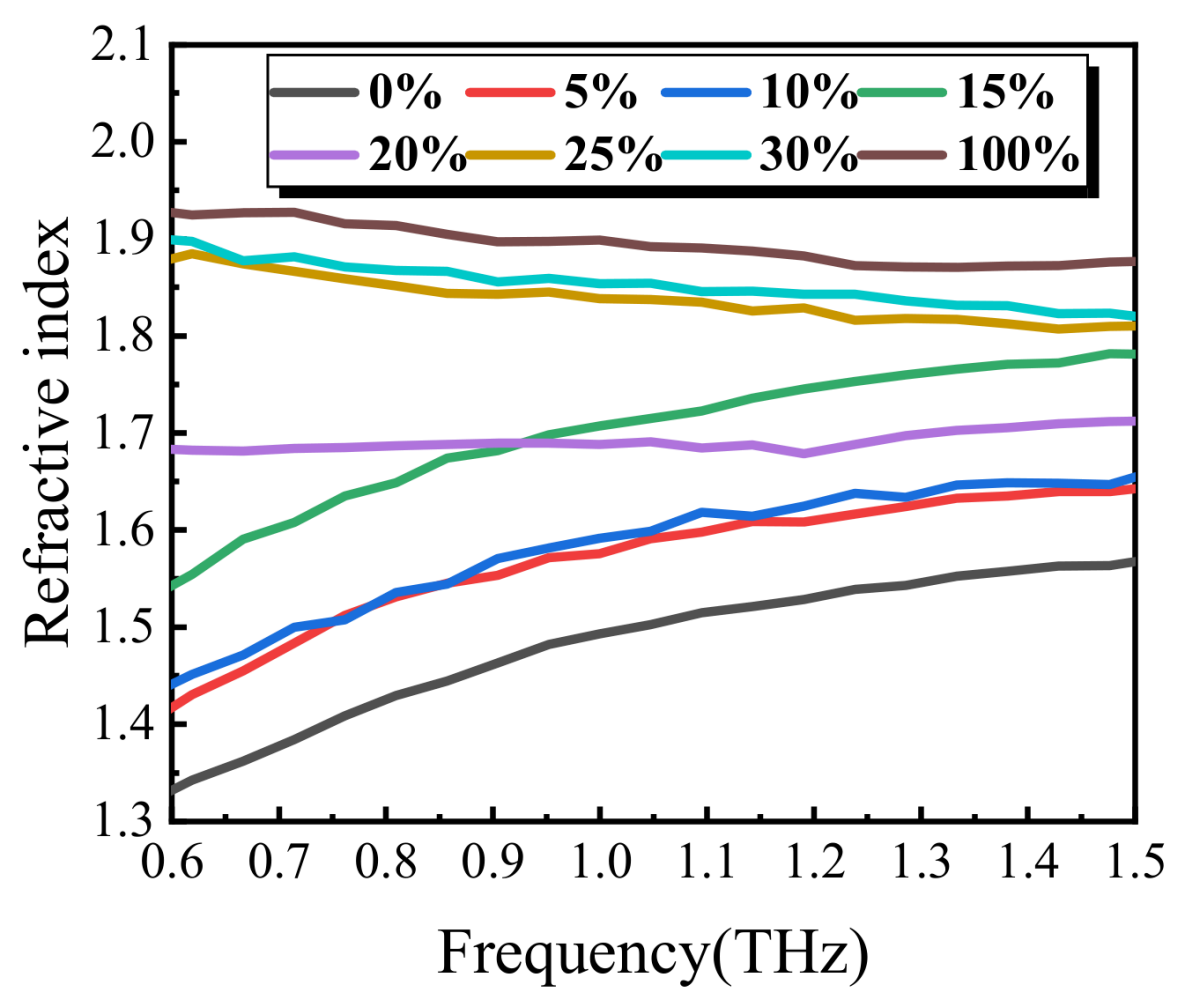
Refractive-index spectra of specimens with different gangue contents.

**Figure 11:**
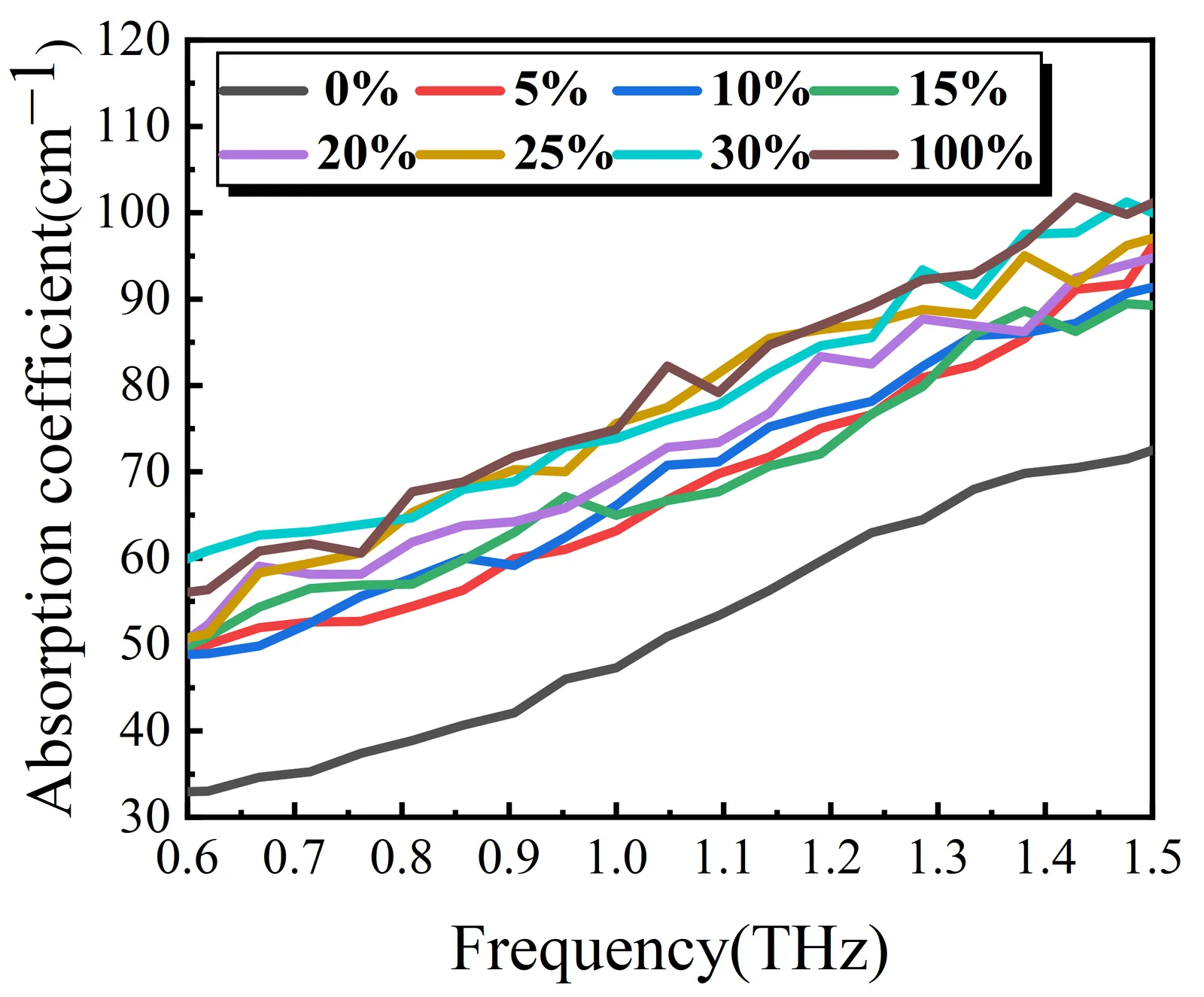
Absorption-coefficient spectra of specimens with different gangue contents.

**Figure 12:**
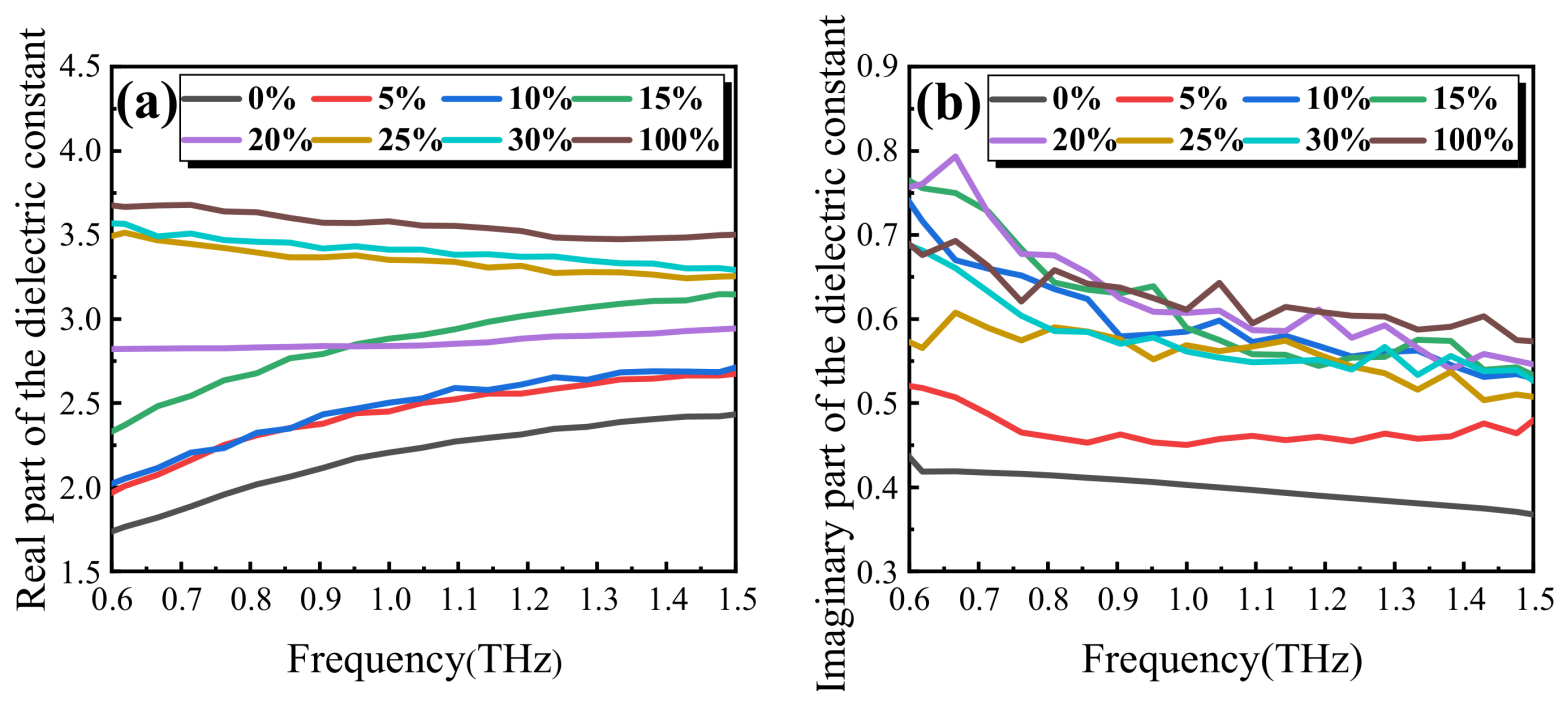
Complex-permittivity spectra of specimens with different gangue contents: (**a**) real part; (**b**) imaginary part.

To characterize the intrinsic electromagnetic response, the refractive index, absorption coefficient, and complex permittivity were calculated from the transmission model (Figure 10, Figure 11 and Figure 12). The optical parameters show clear composition-dependent differences. The refractive index generally increases with gangue content, indicating enhanced polarization. The absorption coefficient also rises, consistent with stronger electromagnetic loss [30,31]. Systematic changes in both the real and imaginary parts of the permittivity further confirm that mixture composition controls the effective dielectric response [32].

These results demonstrate that THz-TDS provides not only time- and frequency-domain observables, but also physically interpretable optical parameters that reflect compositional differences. Together, these features form the basis for multidomain feature fusion and quantitative regression.

### 3.2. Correlation Analysis of Fused Features

**Figure 13:**
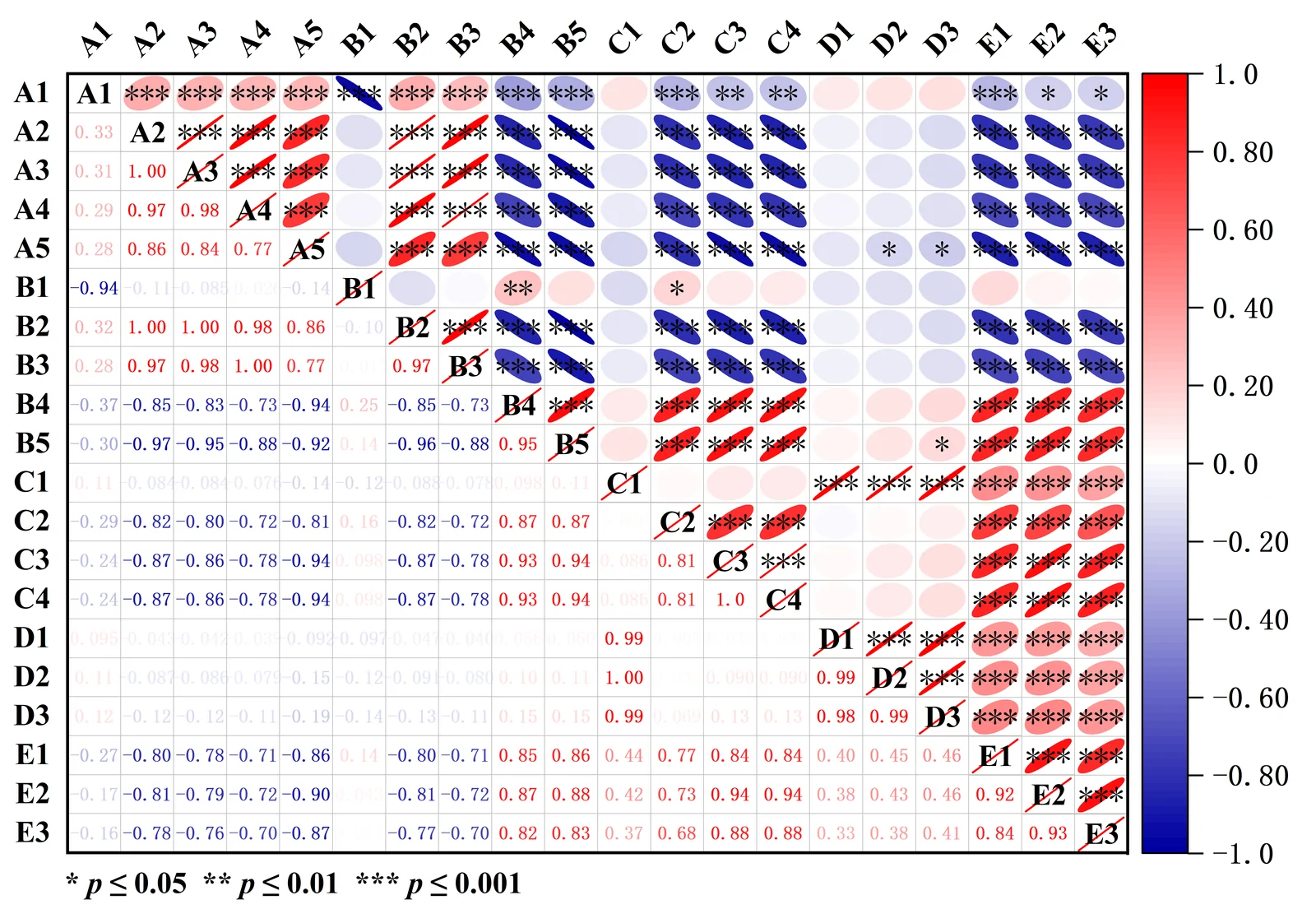
Correlation heat map for the fused time-domain, frequency-domain, and optical-parameter features.

Multidimensional features were extracted from the 175 THz datasets in the 0–30% gangue-content range. Figure 13 presents a correlation heat map in which color intensity represents the magnitude of the correlation coefficient. Ellipse shape and orientation indicate correlation strength and sign: narrower ellipses indicate stronger correlation, with left- and right-leaning orientations denoting negative and positive correlations, respectively.

Statistical significance was assessed by *p*-value testing, using *p* < 0.05, *p* < 0.01, and *p* < 0.001 as significant, highly significant, and extremely significant thresholds. A1–A5 denote time-domain variables; B1–B5 denote frequency-domain variables; C1–C4 denote refractive index at 1.0 THz, absorption coefficients at 0.6 and 1.0 THz, and extinction coefficient at 1.0 THz; and D1–D3 and E1–E3 denote the real and imaginary permittivities at 0.8, 1.0, and 1.2 THz.

Features within the same domain form clear correlation clusters. Most time-domain variables are strongly and significantly positively correlated because they derive from the amplitude, energy, and waveform of the same THz pulse. Mean, root-mean-square, and energy-related variables are particularly correlated, and therefore contain partially overlapping signal-strength information. Frequency-domain variables likewise show strong coupling among spectral amplitude, energy, and centroid. Several frequency-domain variables are also positively correlated with time-domain energy variables, indicating that temporal attenuation is mapped into the spectral response.

The optical parameters show correlations consistent with their physical definitions. Refractive index and the real part of permittivity are strongly positively correlated because both describe energy storage and polarization. Absorption coefficient, extinction coefficient, and the imaginary part of permittivity are also positively correlated because each is related to THz energy loss. Increasing the mineral fraction in gangue enhances conductive and dielectric losses, causing these variables to increase together.

Correlations between the time- and frequency-domain groups are generally weaker than correlations within the optical-parameter group. The three feature domains therefore describe different aspects of the specimens: time-domain variables emphasize delay and attenuation, frequency-domain variables describe spectral energy distribution, and optical parameters represent intrinsic electromagnetic behavior. Combining the domains increases the information content of the model input.

Although some variables are redundant, the feature groups retain substantial complementarity. The correlation analysis therefore supports the use of fused time-domain, frequency-domain, and optical-parameter features for gangue-content regression.

### 3.3. Regression Performance

Table 3 summarizes the five-fold cross-validation results of the five regression models. Among the evaluated methods, EMT-PINN achieved the best overall performance, with an R^2^ of 0.81 ± 0.15, an MAE of 3.17 ± 0.59%, and an RMSE of 5.79 ± 0.21%. The conventional data-driven models showed lower or comparable predictive performance. These results indicate that incorporating the EMT-based physical constraint can improve the quantitative prediction of gangue content from THz-derived features.

### 3.4. Limitations and Future Work

The improved performance of EMT-PINN can be interpreted as the combined effect of multidomain THz feature representation and EMT-based physical regularization. The time-domain and frequency-domain features provide information on pulse delay, attenuation, and spectral energy distribution, whereas the complex permittivity provides a physically interpretable description of the effective dielectric response. During training, the EMT constraint further restricts the predicted gangue fraction according to the experimentally measured dielectric response. Therefore, compared with purely data-driven regression, EMT-PINN simultaneously exploits the statistical information contained in multidomain THz features and prior knowledge of dielectric mixing. Since the present study does not include a fully isolated ablation experiment for separately quantifying the contribution of the feature representation and the EMT constraint, the observed performance improvement should not be attributed exclusively to either component. Instead, the results demonstrate the benefit of their joint integration under the present experimental conditions.

It should be noted that the present study was conducted using a relatively limited number of experimental samples because the preparation of certified coal–gangue mixtures and repeated THz measurements are both labor-intensive and time-consuming. Nevertheless, the incorporation of physical constraints enables EMT-PINN to achieve satisfactory prediction accuracy under small-sample conditions. Although the present experiments were conducted under controlled laboratory conditions to ensure measurement repeatability, this setup represents a necessary methodological validation stage, rather than a direct simulation of the industrial environment. Future work will further validate the proposed framework using larger datasets collected from multiple mining regions.

The use of certified reference materials provides controlled and traceable compositions, which is beneficial for methodological validation. However, these laboratory-prepared mixtures may not fully represent the mineralogical variability, moisture variation, particle-size distribution, and heterogeneity of naturally occurring coal–gangue mixtures. Therefore, the present results demonstrate the feasibility of the proposed framework under controlled conditions, while further validation with independently collected samples is required to assess its generalizability.

## 4. Conclusions

Transmission THz-TDS, multidomain feature fusion, and machine learning were used to predict gangue content in coal–gangue mixtures. The principal findings are summarized as follows.

(1) Increasing gangue content attenuated the THz time-domain amplitude and increased propagation delay. Refractive index, absorption coefficient, extinction coefficient, and complex permittivity also changed systematically, confirming that coal and gangue have distinguishable THz electromagnetic responses.
(2) Time-domain, frequency-domain, and optical-parameter variables contained both redundancy and complementary information. Correlations between refractive index and real permittivity, and among absorption coefficient, extinction coefficient, and imaginary permittivity, were consistent with their physical meaning. Fusing the three domains produced a more informative representation of mixture composition.
(3) EMT-PINN achieved the best average performance (R^2^ = 0.81, MAE = 3.17%, and RMSE = 5.79%) among all evaluated models in five-fold cross-validation, while also providing enhanced physical interpretability through the embedded dielectric mixing constraint. Embedding EMT improved the model’s ability to learn a physically plausible relation between dielectric response and gangue fraction.
(4) Compared with conventional data-driven regression models, the proposed EMT-PINN provides competitive prediction accuracy, together with enhanced physical interpretability, by incorporating dielectric mixing theory into the learning process. The proposed framework demonstrates the potential of combining THz-TDS with physics-informed machine learning for quantitative analysis of heterogeneous coal–gangue mixtures.

## Figures and Tables

**Table 1:** Masses and mixing ratios of coal and gangue powders.

No.	Gangue Content (%)	Gangue Powder (mg)	Coal Powder (mg)
1	0	0	60
2	5	3	57
3	10	6	54
4	15	9	51
5	20	12	48
6	25	15	45
7	30	18	42
8	100	60	0

**Table 2:** Key hyperparameters and architectures of the evaluated machine learning models.

Model	Key Hyperparameters
RF	Number of trees = 100; Minimum leaf size = 4
SVR	Kernel = RBF; C = 10; gamma = 0.1; epsilon = 0.1
GPR	Kernel = ARD-SE; Optimizer = L-BFGS
ANN	Hidden layers = [20, 10]; Activation = ReLU; Optimizer = Adam (lr = 0.001); Batch size = 16; Epochs = 500 (early stopping)
EMT-PINN	Hidden layers = [20, 10]; Activation = tanh; Optimizer = Adam (lr = 0.001); Physics loss weight λ = 0.1

**Table 3:** Performance comparison of different regression models under five-fold cross-validation.

Model	MAE (%)	RMSE (%)	R^2^
RF	4.87 ± 1.03	6.25 ± 0.92	0.72 ± 0.08
SVR	5.18 ± 1.51	6.76 ± 1.64	0.61 ± 0.21
GPR	4.72 ± 0.94	6.05 ± 1.02	0.74 ± 0.11
ANN	5.12 ± 1.25	6.46 ± 1.50	0.64 ± 0.18
EMT-PINN	3.17 ± 0.59	5.79 ± 0.21	0.81 ± 0.15

## Data Availability

The original contributions presented in this study are included in the article. Further inquiries can be directed to the corresponding authors.
